# Evaluation of the Quality of Reporting of Observational Studies in Otorhinolaryngology - Based on the STROBE Statement

**DOI:** 10.1371/journal.pone.0169316

**Published:** 2017-01-06

**Authors:** Martine Hendriksma, Michiel H. M. A. Joosten, Jeroen P. M. Peters, Wilko Grolman, Inge Stegeman

**Affiliations:** 1 Department of Otorhinolaryngology and Head & Neck Surgery, University Medical Center Utrecht, Utrecht, the Netherlands; 2 Brain Center Rudolf Magnus, University Medical Center Utrecht, Utrecht, the Netherlands; Fu Jen Catholic University, TAIWAN

## Abstract

**Background:**

Observational studies are the most frequently published studies in literature. When randomized controlled trials cannot be conducted because of ethical or practical considerations, an observational study design is the first choice. The STROBE Statement (*STrengthening the Reporting of OBservational studies in Epidemiology*) was developed to provide guidance on how to adequately report observational studies.

**Objectives:**

The objectives were 1) to evaluate the quality of reporting of observational studies of otorhinolaryngologic literature using the STROBE Statement checklist, 2) to compare the quality of reporting of observational studies in the top 5 Ear, Nose, Throat (ENT) journals versus the top 5 general medical journals and 3) to formulate recommendations to improve adequate reporting of observational research in otorhinolaryngologic literature.

**Methods:**

The top 5 general medical journals and top 5 otorhinolaryngologic journals were selected based on their ISI Web of Knowledge impact factors. On August 3^rd^, 2015, we performed a PubMed search using different filters to retrieve observational articles from these journals. Studies were selected from 2010 to 2014 for the general medical journals and from 2015 for the ENT journals. We assessed all STROBE items to examine how many items were reported adequately for each journal type.

**Results:**

The articles in the top 5 general medical journals (n = 11) reported a mean of 69.2% (95% confidence interval (CI): 65.8%–72.7%; median 70.6%), whereas the top 5 ENT journals (n = 29) reported a mean of 51.4% (95% CI: 47.7%–55.0%; median 50.0%). The two journal types reported STROBE items significantly different (*p* < .001).

**Conclusion:**

Quality of reporting of observational studies in otorhinolaryngologic articles can considerably enhance. The quality of reporting was better in general medical journals compared to ENT journals. To improve the quality of reporting of observational studies, we recommend authors and editors to endorse and actively implement the STROBE Statement.

## Introduction

Most published studies in literature are observational studies. Observational studies are recognized as a useful insight into identifying best practices and addressing new hypotheses. Sometimes observational studies initiate the need for further investigation and lead to the emergence of randomized controlled trials (RCTs) [1]. Observational studies also offer the opportunity to establish high external validity in a practical setting, which is difficult to achieve in RCTs [2]. While RCTs are being advocated as the gold standard for the evaluation of treatment oriented interventions, well designed observational studies have been shown to provide similar results [3–5]. Because of their design, observational studies are prone to bias, confounding, cause and chance [6]. However, when RCTs cannot be conducted because of ethical or practical considerations, an observational study design is the first choice [7,8]. Clear presentation of its methods, execution and analyses is crucial for its valid implementation in clinical care. Poor reporting of observational studies can further reduce their usefulness.

Therefore, in 2004 [9] the Strengthening the Reporting of Observational Studies in Epidemiology (STROBE) Statement was introduced to improve the quality of reporting of observational studies. The aim of the STROBE statement is to increase transparency in reporting [9]. A checklist made of 22 items was constructed in order to assess the quality of reporting of observational studies. The STROBE Statement is currently being endorsed by a growing number of biomedical journals [10].

To our knowledge, this quality of reporting in otorhinolaryngology has never been assessed before. In otorhinolaryngology, observational studies are an often used study design. Therefore, our objective was to assess the quality of reporting of observational research in otorhinolaryngologic literature using the STROBE Checklist. As a second objective, we aimed to compare the quality of reporting between the top 5 otorhinolaryngologic journals and the top 5 general medical journals, as a benchmark for presumably high quality of reporting. Finally, based on our findings, recommendations were forged in order to improve the reporting of observational studies in otorhinolaryngology.

## Methods

### Journals

We selected the top 5 medical journals (*New England Journal of Medicine* (NEJM), *The Lancet*, *Journal of the American Medical Association* (JAMA), *British Medical Journal* (BMJ) and *PLOS Medicine* (PLOS Med)) and the top 5 Ear Nose Throat (ENT) journals (*Rhinology*, *Hearing Research* (Hear Res), *Ear & Hearing* (Ear Hear), *Head & Neck* and *Journal of the Association for Research in Otolaryngology* (JARO)) based on their ISI Web of Knowledge impact factors. **Table 1** presents the ISI Web of Knowledge impact factors of 2012 for general medical journals and of 2014 for ENT journals (www.webofknowlegde.com, date of access August 3^rd^, 2015).

**Table 1:** Top 5 general medical journals and ENT journals impact factors and STROBE endorsement.

Rank	Title	Impact factor*	Endorse STROBE?
**General medical journals**			
1	New England Journal of Medicine (NEJM)	51.658	No
2	The Lancet	39.060	Yes
3	Journal of the American Medical Association (JAMA)	29.978	No
5	The British Medical Journal (BMJ)	17.215	Yes
7	PLOS Medicine (PLOS Med)	15.976	Yes
**ENT Journals**			
1	Rhinology	3.761	No
2	Hearing Research (Hearing Res)	2.968	No
3	Ear & Hearing (Ear Hear)	2.842	No
4	Head & Neck (Head Neck)	2.641	No
5	Journal of the Association for Research in Otolaryngology (JARO)	2.598	No

* Source: ISI Web of Knowledge. For general medical journals impact factors of 2012. For ENT journals impact factors of 2014. Both assessed on August 3^rd^, 2015.

ENT = Ear Nose Throat.

In addition, we evaluated the ‘Instruction to authors’ section on the websites of the included journals to check if they endorsed the STROBE statement (see also **Table 1**).

### Search

We performed a PubMed search on August 3^rd^ 2015 using several search syntaxes. First, to retrieve only observational studies, a syntax developed by the Scottish Intercollegiate Guidelines Network was used [11]. Second, an adapted version of the Cochrane ENT search syntax was used to retrieve otorhinolaryngologic articles [12]. Third, a filter was used to retrieve articles published in the top 5 general medical journals (**Table 1**) and articles published in the top 5 ENT journals (**Table 1**) separately. Fourth, a date restriction filter was applied. To retrieve sufficient otorhinolaryngologic articles published in general medical journals, we searched from January 1^st^, 2010 until December 31^st^, 2014. We did not search for articles earlier than 2010, since the STROBE Statement was first published in 2007. Hereby, we allowed for sufficient time for implementation of the STOBE Statement by study authors. Naturally, it is easier to retrieve otorhinolaryngologic articles in ENT journals, so for articles published in ENT journals we applied a date restriction from January 1^st^, 2015 until August 3^rd^, 2015. Finally, a combination of search syntaxes was made to retrieve observational studies from general medical journals and ENT journals respectively (see **S1 File**).

### Study selection

Two authors (MH and MHMAJ) independently assessed titles, abstracts and full texts of the retrieved articles to check if the study was indeed an observational study and if it was conducted in the otorhinolaryngologic field. To be considered an observational study, studies must have had a cross-sectional, cohort or case control design. To be considered as a study in the otorhinolaryngologic field, studies must have assessed patient populations generally treated by ENT physicians or procedures generally performed by ENT physicians, including procedures performed by head and neck surgeons. Discrepancies between the two reviewers were discussed until consensus was reached.

### Strobe statement adherence

To score the quality of reporting, the most recent version of the STROBE statement was used [9]. The included articles were read and scored independently by two authors (MH and MHMAJ). We evaluated the items of the STROBE checklist that were adequately reported. The total number of items on the STROBE checklist is 34 (subitems included). Some items (6a, 6b, 12d, 14c, 15) are specific for some study designs only (e.g. cohort or case control). Consequently, if an item was not applicable for the study design, it was scored as ‘not applicable’. For a more detailed description of the requirements to score ‘adequately reported’, see **S2 File**.

Because of an imbalance in the number of articles per journal category, we scored one article from the general medical journals for every three articles from the ENT journals, so a possible learning effect in use of the STROBE checklist was distributed evenly across both journal categories. Differences in opinion were discussed until consensus was reached.

### Data analysis

We divided the number of adequately reported items by the total number of applicable items, which resulted in a proportion of adequately reported items. A higher proportion reflects that the item was reported more adequately. Mean, medians and 95% confidence intervals (CI) were calculated per item.

The 2-tailed Mann Whitney U test for two independent samples was used to test if there was a statistically significant difference between the STROBE scores for articles published in the top 5 general medical journals and in the top 5 ENT journals.

Furthermore, the interobserver agreement (Cohen’s kappa) was calculated to determine if there were large differences in the scoring of items between the two authors.

Statistical tests were performed using SPSS v21 statistics package. A *p*-value of < .05 was considered statistically significant.

## Results

### Search

**Fig 1:**
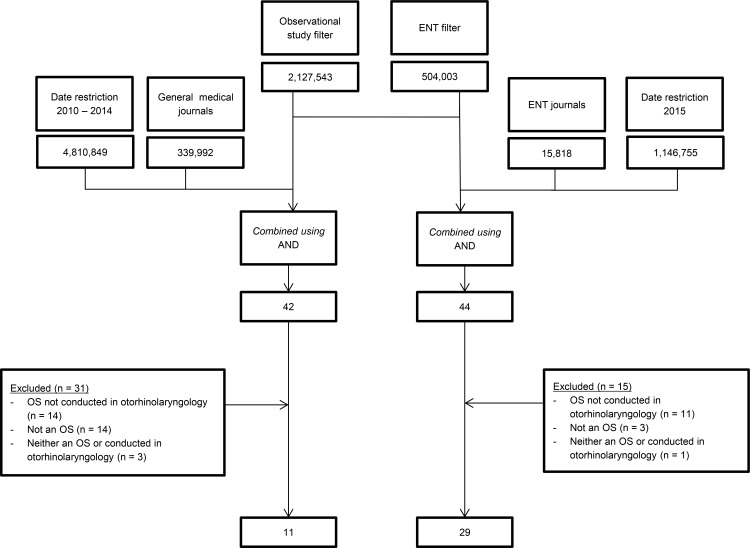
Flowchart of search. Date of search: August 3^rd^, 2015. For complete search syntax, see **S1 File**. ENT = Ear, Nose, Throat, OS: observational study, N = number.

The search process is shown in **Fig 1**. The combined search syntaxes yielded 42 articles from general medical journals and 44 from ENT journals.

### Study selection

Of the 42 articles in the general medical journals, 14 articles were not considered otorhinolaryngologic articles, 14 articles were considered not to be an observational study and 3 articles were neither considered an otorhinolaryngologic nor an observational study. Finally, we included 11 observational studies on otorhinolaryngologic topics from the general medical journals.

Of the 44 articles in the ENT journals, 11 articles were not considered to be an observational study, 3 articles were not considered otorhinolaryngologic articles, and 1 article was considered neither an otorhinolaryngologic nor an observational study. Finally, we included 29 observational studies on otorhinolaryngologic topics from ENT journals.

### STROBE Statement adherence

**Fig 2:**
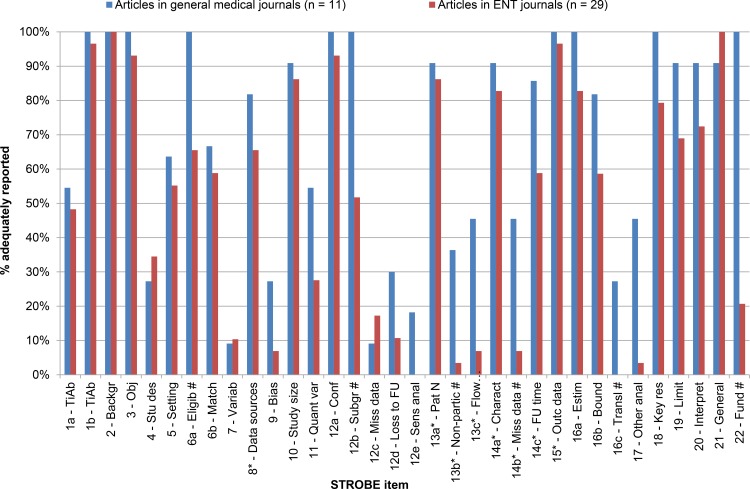
Adequately reported STROBE items. For an overview of all STROBE items see **S2 File**. Items highlighted with a # are statistically significant between journal categories. ENT = Ear, Nose, Throat.

The 11 articles published in general medical journals (NEJM = 1, Lancet = 1, JAMA = 4, BMJ = 2, PLOS Med = 3) reported a mean of 69.2% (95% CI: 65.8%–72.7%; median 70.6%) of STROBE items adequately. The 29 articles published in ENT journals (Rhinology = 1, Hear Res = 0, Ear & Hearing = 8, Head & Neck = 20 and JARO = 0) reported a mean of 51.4% (95% CI: 47.7%–55.0%; median 50.0%) of STROBE items adequately. The exact percentage of adequately reported STROBE items for studies published in general medical journals and ENT journals are presented in **Table 2**. A graphic illustration is provided in **Fig 2**.

**Table 2:** data table of Fig 2. Percentage of adequately reported STROBE items per journal category.

**Item**	**1a - TiAb**	**1b - TiAb**	**2 - Backgr**	**3 - Obj**	**4 - Stu des**	**5 - Setting**	**6a - Eligib**	**6b - Match**	**7 - Variab**	**8 - Data sourc**	**9 - Bias**	**10 - Study size**	**11 - Quant var**	**12a - Conf**	**12b - Subgr**	**12c - Miss data**	**12d - Loss to FU**
Articles in general medical journals (n = 11)	55%	100%	100%	100%	27%	64%	100%	67%	9%	82%	27%	91%	55%	100%	100%	9%	30%
Articles in ENT journals (n = 29)	48%	97%	100%	93%	34%	55%	66%	59%	10%	66%	7%	86%	28%	93%	52%	17%	11%
*p*-value Chi2 (2-tailed)	1,000	1,000	N/A	1,000	1,000	0,730	**0,038**	1,000	1,000	0,451	0,117	1,000	0,147	1,000	**0,004**	1,000	0,310
**Item**	**12e - Sens anal**	**13a - Pat N**	**13b - Non-partic**	**13c - Flow Diagram**	**14a - Charact**	**14b - Miss data**	**14c - FU time**	**15 - Outc data**	**16a - Estim**	**16b - Bound**	**16c - Transl**	**17 - Other anal**	**18 - Key res**	**19 - Limit**	**20 - Interpret**	**21 - General**	**22 - Fund**
Articles in general medical journals (n = 11)	18%	91%	36%	45%	91%	45%	86%	100%	100%	82%	27%	45%	100%	91%	91%	91%	100%
Articles in ENT journals (n = 29)	0%	86%	3%	7%	83%	7%	59%	97%	83%	59%	0%	3%	79%	69%	72%	100%	21%
*p*-value Chi2 (2-tailed)	0,071	1,000	**0,015**	**0,011**	1,000	**0,011**	0,352	1,000	0,298	0,270	**0,017**	**0,004**	0,162	0,233	0,399	0,275	**0,000**

**Legend:** Yellow cell and bold font = significant difference for quality of reporting between journal types (Chi2, p < 0.05). For an overview of all STROBE items see **S2 File**. ENT = Ear Nose Throat.

We compared the difference in reporting individual items between observational studies published in general medical journals and ENT journals. The ENT journals reported several items inadequately more frequently than general medical journals. The difference in reporting between journal categories was statistically significant for 8 out of 34 items (highlighted with a # in **Fig 2**). First, less than 25% of the articles published in ENT journals reported funding and the role of the funders, whereas this was reported in 100% of the articles published in general medical journals (item 22). Second, several other items were scored significantly better in general medical journals than in ENT journals. Item 6a (eligibility criteria and the sources of methods of selection of participation) and item 8 (data sources and measurement for each variable of interest) were scored better by general medical journals, as well as item 12b (methods used to examine subgroups and interaction). Likewise for items 13a, 13b and 13c (reporting of numbers of individuals at each stage of the study, reasons for non-participation at each stage and the use of a flow diagram). Also items 14a, 14b and 14c (descriptive data of the study participants) and at last item 15 (the outcome data) were all scored significantly better in general medical journals.

Besides these differences, both types of journals scored insufficient on several other items. For example, both journal categories scored poorly in reporting the variables (item 7); also both categories of journals reported less than 20% on items 9, 12c, 12d and 12e. Moreover, none of the articles published in ENT journals reported sensitivity analyses (item 12e) or translated estimates of relative risk into absolute risk (item 16c).

The overall interobserver agreement kappa is 0.64. A kappa score between 0.61 and 0.80 means there is a good agreement [13]. **S3 File** shows the interobserver agreement per STROBE item for both journal categories, as well as both journal categories together. Items scored with the most discrepancy between the two authors, were the defining of outcomes (item 7), the handling of quantitative variables (item 11), the reporting of individuals at each stage of the study (item 13a), the study characteristics of participants (item 14a), the reporting of unadjusted estimates (item 16c), the overall interpretation of results (item 20) and the generalizability (item 21).

## Discussion

To the best of our knowledge, this study is the first to assess the quality of reporting of observational studies in otorhinolaryngologic literature. Furthermore, we made a comparison between the top 5 general medical journals and the top 5 ENT journals, by using the STROBE checklist. This study shows a substantial difference in the quality of reporting between ENT journals and general medical journals.

### Interpretation of results

First, notable differences were reported regarding the eligibility criteria (item 6a). Eligibility criteria help readers understand the applicability and generalizability of the reported results. In observational studies, these characteristics are often not adequately reported [14].

Second, sub-analyses are only reported in about 50% of the top 5 ENT journals. Being one of the items of the STROBE checklist, there is debate about this item (item 12b) [15,16]. The reporting of sub-analyses allows one to examine whether effects or associations differed across groups [17].

Third, missing data is common in observational research (item 14b). It is essential to describe for which variable of interest data was missing, because (in)voluntary exclusion of patients can lead to a distorted image in the results.

Fourth, other analyses were only reported in 3% of the articles published in ENT journals (item 17). However, these analyses are important, because they may address specific subgroups, display potential interaction between risk factors, calculate attributable risks or use alternative definitions of study variables in sensitivity analyses [17].

Fifth, almost 80% of the ENT journals do not report funding (item 22), although there are strong associations between the source of funding and conclusions [18,19]. It is therefore important to be transparent about financial support, as specific types of sponsorships may be associated with positive study results [20].

Sixth, the use of a flow diagram is an easy way to display extensive information in a compact design (item 13c); 7% of the articles published in ENT journals made use of a flow diagram compared to 45% of the articles published in general medical journals.

Seventh, reasons for non-participation (item 13b) was reported in 3% of the ENT journals. This item lets readers judge whether the study population was representative of the target population [17].

Last, translating estimates of relative risk into absolute risk (item 16c) was not reported in any of the ENT articles. However, in some cases this item may not be relevant.

As for the top 5 general medical journals, 11 items scored particular lower than expected, i.e. less than 50%; items 4, 7, 9, 12c, 12d, 12e, 13b, 13c, 14b, 16c and 17. Of these, 6 items (item 4, 7, 9, 12c, 12d and 12e) scored similar poor compared to the top 5 ENT journals (no statistical difference).

### Comparison with literature

In other research fields, like ophthalmology and hand surgery, items like bias (item 9), the explanation of missing data (item 12c), use of a flow-diagram (item 13c) and indicating missing data for each variable of interest (item 14b) scored similarly insufficient to the articles published in ENT journals [21,22]. Items which scored better in these research fields were presenting key elements of study design early in the paper (item 4), defining all outcomes, exposures, predictors, potential confounders and effect modifiers (item 7), describing loss to follow-up/matching of cases and controls/sampling (item 12d) and displaying characteristics of study participants (item 14c). Only item 10, the explanation of how study size was arrived at, was scored better in the articles published in ENT journals in our sample. In another study, Delaney et al. evaluated platelet transfusion studies according to the STROBE criteria [23]. They also scored missing data as reported inadequately, in accordance with our results. Unfortunately, an average compliance score was not calculated [23]. Parsons et al.[24] evaluated general orthopedic journals and found an overall average compliance to the STROBE checklist of 58% (95% CI: 56%–60%), close to our findings. There were 9 items that scored similarly poor (items 9, 12c, 12d, 12e, 13b, 13c, 14b and 16c). It was unclear whether the journals in which the included articles were published endorsed the STROBE Statement. Since the articles included by Parsons et al. were selected from the years 2005 to 2010, active endorsement was not entirely possible, as the STROBE Statement was first published in 2007.

Similar results were seen in the research field of plastic surgery, where the 9 items mentioned above along with item 17 scored similarly poor in reference to our results [25]. This study also suggested that reporting could possibly be improved by making the STROBE checklist mandatory at submission [25].

One other study showed that the quality of reporting of confounding improved in some aspects, but the authors concluded that the overall quality remains suboptimal [26]. Quantitative bias analysis (item 9) was scored very rarely, which we also observed.

Two reporting guidelines (Consolidated Standards of Reporting Trials (CONSORT) and Preferred Reporting Items for Systematic Reviews and Meta-analyses (PRISMA)) have been published before the STROBE Statement. Several studies found that the endorsement of the CONSORT and PRISMA Statements led to a better quality of reporting [27–34]. Two other articles evaluating the CONSORT Statement concluded that active endorsing would further increase the quality of reporting [35,36]. Unfortunately, no studies have been conducted evaluating the STROBE Statement for multiple disciplines simultaneously or investigating the implementation of the STROBE Statement. For better applicability of the STROBE Statement, some papers have adjusted the STROBE statement to their specific research field [37,38].

### Methodological considerations

Strengths of our study include the independent assessment of all articles by two authors. Besides, to accumulate the learning effect in reference to the imbalance in the number of articles per journal type, we scored one article from general medical journals for every three articles from ENT journals. All details of our search were clear and transparent and our search can therefore easily be reproduced. Furthermore, some of the authors were involved in the assessment of the PRISMA and CONSORT Statement checklists [39,40]. This scientific context and experience could have improved the overall quality of our study. Additionally an inter-observer agreement (Cohen’s kappa) was calculated to determine which items were often disputed (these items deserve extra attention when assessing, and possibly even rephrasing by the STROBE workgroup, because they may be hard to interpret).

However, our study also has limitations. First, scoring of items remains a subjective task, with differences between observers. Several items were prone to discussion between the scoring authors. However, our analysis showed that we reached good interobserver agreement (Cohen’s kappa 0.64). Second, only eleven studies were found for the top 5 general medical journals. This small sample size makes it more difficult to draw appropriate conclusions for this journal category. Third, the inclusion periods (top 5 general medical journals: January 1^st^, 2010 –December 31^st^, 2014; top 5 ENT journals January 1^st^, 2015 –August 3^rd^, 2015) of both journals types were not identical. As expected, less otorhinolaryngologic articles were published in general medical journals than in ENT journals. Even with a broader time frame in the search syntax for articles from general medical journals, this still resulted in less articles (n = 11) than the number of articles included from ENT journals (n = 29). However, retrieving earlier studies would not lead to a more accurate comparison, since the STROBE Statement was first published in 2007. Consequently, earlier studies would not have had time to implement the STROBE Statement. On the other hand, we did not broaden our date restriction for ENT journals, since 29 articles form a good sample to base conclusions on. Including otorhinolaryngologic articles from ENT journals from 2010–2014 would result in many more articles to score, leading to incomparable samples. Moreover, including more articles published in ENT journals would probably not have changed conclusions. Finally, by choosing the most recent articles published in ENT journals, we would expect maximum improved quality of reporting, because more time was available to implement the STROBE Statement. It would be interesting to evaluate the quality of reporting over a period of time. However, eleven articles from five years do not provide enough statistical power for such a comparison.

### Recommendations

Clear and transparent reporting in research papers facilitates clinicians and researchers that they can evaluate the validity of articles. The STROBE Statement was developed to aid researchers to report their observational studies adequately. Therefore, we think it is highly plausible that using the STROBE Statement will improve the quality of reporting. We recommend authors of otorhinolaryngologic articles to use the STROBE Statement and recommend editors of otorhinolaryngologic journals to actively endorse this statement in their guidelines for authors.

## Conclusion

Current quality of reporting of observational studies in otorhinolaryngologic journals is suboptimal. According to the STROBE Statement checklist, the reporting of otorhinolaryngologic observational studies is significantly better in articles published in general medical journals (2010–2014) than in articles published in ENT journals (2015). We recommend authors of otorhinolaryngologic articles to use the STROBE Checklist to help them to improve the quality of reporting. Furthermore, we suggest editors of ENT journals to actively endorse the STROBE Statement in their submission process.

## Supporting Information

S1 File Search Syntaxes. Date of search: August 3^rd^, 2015. We used a study syntax developed by the Scottish Intercollegiate Guidelines Network [11]. An adapted version of the Cochrane ENT search syntax was used to retrieve otorhinolaryngologic [12] articles. Finally, a date restriction was applied from January 1^st^, 2010 until December 31^st^, 2014 for the top 5 general medical journals and from January 1^st^, 2015 until August 3^rd^, 2015 for the top 5 ENT journals.
(DOCX)

S2 File Explanation of reported items of the STROBE checklist. The original STROBE Statement can be assessed by visiting the STROBE website [10] or see Von Elm et al. [9]. For an explanatory and elaborated view see Vandenbroucke et al. [17]. Items were either scored as ‘adequately reported’, ‘inadequately reported’ or ‘not applicable’. Five items of the STROBE Statement are specific for study design (6a, 6b, 12d, 14c, 15). If any of these items were not applicable for the study design, it was scored as ‘not applicable’. ‘Not applicable’ items were not added to the amount of items to score.
(DOCX)

S3 File Kappa per STROBE item. The interobserver agreement for each STROBE item was calculated for both journal categories, as well as both journal categories together. For an explanation of scale division for the interobserver agreement, see Altman [13].
(DOCX)
