# Supplementary material for: Evaluation of the Quality of Reporting of Observational Studies in Otorhinolaryngology - Based on the STROBE Statement
Source: PLoS One. 2017 Jan 6;12(1):e0169316. doi: 10.1371/journal.pone.0169316 (PMC5217955; doi:10.1371/journal.pone.0169316)
Supplement: S2 File — The original STROBE Statement can be assessed by visiting the STROBE website [10] or see Von Elm et al. [9]. For an explanatory and elaborated view see Vandenbroucke et al. [17]. Items were either scored as ‘adequately reported’, ‘inadequately reported’ or ‘not applicable’. Five items of the STROBE Statement are specific for study design (6a, 6b, 12d, 14c, 15). If any of these items were not applicable for the study design, it was scored as ‘not applicable’. ‘Not applicable’ items were not added to the amount of items to score. (DOCX) [file pone.0169316.s002.docx]

**Supporting Information 2. Adequately reported items of the STROBE checklist**

| **Item** | | **Criteria to score as adequately reported** |
| --- | --- | --- |
| **Title and abstract** | | |
| **1a** | Study design | The study design in the abstract or title containing cohort, case-control or cross-sectional was described. |
| **1b** | Abstract | An informative and balanced summary of the paper was provided. |
| **Introduction** | | |
| **2** | Background | Explanation about the specific (scientific) background was given. |
| **3** | Objective / hypotheses | A specific objective or hypothesis was mentioned. |
| **Methods** | | |
| **4** | Study design | The study design was described early in the methods section containing cohort, case-control or cross-sectional. |
| **5** | Setting | The setting, locations and relevant dates including periods of recruitment, data collection, follow-up and exposure were described. |
| **6a** | Participants, selection criteria | Depending on the study design sources and methods of selection of participants or sources and methods of case ascertainment and control selection were described. |
| **6b** | Matching criteria | *Not applicable for cross-sectional studies*  Matching criteria and number of exposed / unexposed or number of controls per case were given. |
| **7** | Variables | All outcomes, predictors, potential confounders and effect modifiers were defined. |
| **8** | Data sources / measurement | For each variable of interest sources of data and details of methods of assessments were given. |
| **9** | Bias | The methods to assess risk of bias across the study were described. |
| **10** | Study size | It was explained how study size was created. |
| **11** | Quantitative variables | The method of how quantitative variables were categorized was described. |
| **12a** | Statistical methods | The statistical method(s) to analyse the data were provided. |
| **12b** | Subgroups / interactions | The statistical method(s) to examine subgroups and interactions were provided. |
| **12c** | Missing data | It was explained how missing data were handled. |
| **12d** | Follow-up / matching or sampling strategy | It was described how loss to follow-up was addressed, how matching of cases and controls was addressed or how reporting of analytical methods was done while taking account of sampling strategy, depending on the study design. |
| **12e** | Sensitivity analysis | A sensitivity analysis was described. |
| **Results** | | |
| **13a** | Participants | Numbers of individuals at each stage of the study were provided. |
| **13b** | Non-participation | Reasons for non-participation were provided for each stage. |
| **13c** | Flow diagram | A flow diagram was used. |
| **14a** | Descriptive data | Characteristics of study participants (e.g. demographic, clinical or social) and optionally exposure and potential confounders was given. |
| **14b** | Missing data | Number(s) of participants with missing data for variables of interest were given. |
| **14c** | Follow-up time | *Not applicable for case-control or cross-sectional studies*  Only for cohort studies: follow-up time was described. |
| **15** | Outcome data | Depending on the study design, numbers of outcome events or summary measures (over time of exposure) were described. |
| **16a** | Main results | Unadjusted estimates and their precision were given. |
| **16b** | Category boundaries | Category boundaries were reported when continuous variables were categorized. |
| **16c** | Relative risk into absolute risk | Translating estimates of relative risk into absolute risk for a meaningful time period was reported. |
| **17** | Additional analyses | Sensitivity analyses or other extra analyses were reported. |
| **Discussion** | | |
| **18** | Summary key results | The discussion section started with a summary of the key results with reference to the study objectives. |
| **19** | Limitations | Limitations or potential sources of bias of the current study were described. |
| **20** | Overall interpretation | The overall interpretation of results considering objectives, limitations and results from similar studies or other relevant evidence were described. |
| **21** | Generalizability | The generalizability of the study results was described. |
| **Other information** | | |
| **22** | Funding and role of funders | Sources of funding and role of the funders of the current study were provided. |

**Legend:**

Based on STROBE checklist (Von Elm 2007) and suggestions in the *Explanation and Elaboration* paper (Vandenbroucke 2007).

Items were scored as either ‘adequately reported’ or ‘inadequately reported’; there was no category ‘partially adequately reported’. If an item was not applicable for that study design, it was scored as ‘not applicable’.
