# Supplementary material for: Evaluation of the Quality of Reporting of Observational Studies in Otorhinolaryngology - Based on the STROBE Statement
Source: PLoS One. 2017 Jan 6;12(1):e0169316. doi: 10.1371/journal.pone.0169316 (PMC5217955; doi:10.1371/journal.pone.0169316)
Supplement: S3 File — The interobserver agreement for each STROBE item was calculated for both journal categories, as well as both journal categories together. For an explanation of scale division for the interobserver agreement, see Altman [13]. (DOCX) [file pone.0169316.s003.docx]

**Supporting Information 3: Kappa per STROBE item**

| **Quality of reporting** | **1a - TiAb** | **1b - TiAb** | **2 - Backgr** | **3 - Obj** | **4 - Stu des** | **5 - Setting** | **6a - Eligib** | **6b - Match** | **7 - Variab** | **8* - Data sources** | **9 - Bias** | **10 - Study size** | **11 - Quant var** | **12a - Conf** | **12b - Subgr** | **12c - Miss data** | **12d - Loss to FU** | **12e - Sens anal** |
| --- | --- | --- | --- | --- | --- | --- | --- | --- | --- | --- | --- | --- | --- | --- | --- | --- | --- | --- |
| Articles in general medical journals (n = 11) | 0,820 | NA | NA | 0,000 | 0,744 | 0,441 | NA | 1,000 | 0,020 | 0,560 | 0,744 | 1,000 | 0,098 | NA | 0,000 | -0,100 | -0,174 | 1,000 |
| Articles in ENT journals (n = 29) | 0,791 | 1,000 | NA | 0,651 | 0,721 | 0,316 | 0,212 | 0,217 | 0,249 | 0,180 | 1,000 | 0,628 | 0,129 | 0,346 | 0,371 | 1,000 | 1,000 | NA |

| **Quality of reporting** | **13a* - Pat N** | **13b* - Non-partic** | **13c* - Flow Diagram** | **14a* - Charact** | **14b* - Miss data** | **14c* - FU time** | **15* - Outc data** | **16a - Estim** | **16b - Bound** | **16c - Transl** | **17 - Other anal** | **18 - Key res** | **19 - Limit** | **20 - Interpret** | **21 - General** | **22 - Fund** |
| --- | --- | --- | --- | --- | --- | --- | --- | --- | --- | --- | --- | --- | --- | --- | --- | --- |
| Articles in general medical journals (n = 11) | 0,000 | 0,476 | 0,441 | 0,020 | 0,621 | 0,222 | NA | NA | 0,744 | 0,000 | 0,353 | NA | 1,000 | 0,000 | 0,000 | NA |
| Articles in ENT journals (n = 29) | 0,203 | 0,341 | 1,000 | 0,103 | 0,651 | 0,486 | 0,463 | 0,449 | 0,580 | -0,055 | -0,061 | 0,208 | 0,754 | 0,171 | -0,048 | 0,703 |

**Legend:** Overall interobserver agreement kappa is 0.64. Items with the most discrepancy between authors are displayed in yellow typeface. For an overview of all STROBE items, see **S2 File.**
